# Poster Session I - A25 INFECTION WITH THE CROHN’S DISEASE PATHOBIONT *E. COLI*-LF82 REVEALS MITOCHONDRIAL DNA RELEASE AND CGAS-STING ACTIVATION

**DOI:** 10.1093/jcag/gwaf042.025

**Published:** 2026-02-13

**Authors:** A Mohan, A Wang, D McKay, T Shutt

**Affiliations:** University of Calgary, Calgary, AB, Canada; University of Calgary, Calgary, AB, Canada; University of Calgary, Calgary, AB, Canada; University of Calgary, Calgary, AB, Canada

## Abstract

**Background:**

We have shown that the Crohn’s disease-associated adherent invasive *E. coli* (AIEC) (strain LF82) fragments the mitochondrial network of cancer-derived colon epithelial cells, colon organoids, and primary colon epithelial cells. In other situations where significant mitochondrial dysfunction occurs there is mtDNA escape into the cytosol causing activation of inflammatory pathways such as cGAS-STING. Recent studies have shown activation of cGAS-STING as a potential disease modifier in inflammatory bowel disease (IBD). Therefore, we sought to investigate if mtDNA escape occurs in AIEC-LF82 infected epithelia and if this leads to upregulation of inflammatory pathways.

**Aims:**

Assess mtDNA release following infection with *E. coli*-LF82 in a primary colonocyte cell line as well as subsequent activation of mtDNA-sensing inflammatory pathways.

**Methods:**

FHC epithelia were infected with *E. coli*-LF82 or the commensal *E. coli*-HB101 at multiplicity of infection (MOI) of 100 or uninfected as a control. To assess mtDNA escape, FHCs were stably transduced with the mt-HI-NESS construct to fluorescently label mtDNA and co-stained with MitoView Fix 640. The cells were blindly assessed via live-cell imaging using an IX83 Spin SR microscope to observe mitochondrial morphology and mtDNA localization. For inflammatory pathway studies, FHCs were treated with the cGAS inhibitor G140 (pre-treatment for 16h, 20 µM), the STING inhibitor H-151 (co-treatment, 4 µg/mL) or vehicle and left uninfected or infected with *E. coli*-LF82 at MOI=100 for 8h. *CXCL10, IL6, IL8,* and *CXCL11* expressions were assayed via qRT-PCR.

**Results:**

Epithelia infected with *E. coli*-LF82 but not *E. coli*-HB101 displayed dramatic fragmentation of their mitochondrial network coinciding with extra-mitochondrial mtDNA nucleoids beginning 6h post-infection. Additionally, we found that AIEC-LF82 infection results in increased transcription of pro-inflammatory chemokines and cytokines *CXCL10, CXCL11, IL6*, and *IL8* 8h post-infection. The AIEC-LF82 induced transcription of inflammatory cytokines *CXCL10*, and *CXCL11* was significantly attenuated by pharmacological inhibition of cGAS or STING, while *IL6* and *IL8* mRNA expressions were only partially reduced and remain above control levels (n = 2-3).

**Conclusions:**

These data suggest AIEC-LF82 infection causes mtDNA release in colon epithelial cells and increases the expression of pro-inflammatory cytokines via the cGAS-STING pathway. There appears to be two components to the response, one of which is cGAS-STING dependent and the other which is increased but not completely dependent on cGAS-STING activation, potentially illustrating a mechanism by which cGAS-STING activation is a disease modifier in IBD pathogenesis. As a result, this may suggest that inhibition of cGAS-STING as a therapeutic may be applicable in cases of IBD with mitochondrial dysfunction.

**Funding Agencies:**

CAG, CIHRTRIANGLE

